# New Appliances and Things Medical

**Published:** 1900-10-13

**Authors:** 


### NEW APPLIANCES AND THINGS MEDICAL.

[We shall be glad to receive, at our Office, 28 & 29 Southampton Street, Strand, London, W.C., from the manufacturers, specimens of all new preparations and appliances which may be brought out from time to time.]

### OXO.

(Liebig's Extract of Meat Company, Fenchurch Avenue, London, E.C.)

Oxo is a fluid beef, which, it is hoped by the manufacturers, will fulfil a want long felt by those who cannot afford the luxury of home-made beef tea, or the expensive substitutes on which they have had previously to depend. Oxo is sold in two-ounce bottles, sufficient to make sixteen teacups of very excellent beef tea. The price for each cup works out at very little more than one halfpenny. The flavour of oxo is very different from what we have long been accustomed to, and, if we may add the expression, put up with, in many of the meat extracts with which we are familiar. The flavouring is delicate and not excessive, and, having made trial of this new fluid beef, we are willing to admit that we found it very much to our taste. We thoroughly recommend it to the notice of those who want a really cheap and excellent preparation of this kind.

### THE "HODGKINSON" ACETYLENE SURGICAL LAMP. BIRCH TAR AND SULPHUR SOAP, AND EMOLLIENT BATH POWDER.

(Charles Midgley & Co., 23 St. Anne's Square, Manchester.)

The "Hodgkinson" acetylene lamp is a neat and simple contrivance for illuminating the throat, eye, ear, &c., during clinical examination at the bedside, or at other times when gas or electric light are not obtainable. The adjustable stand can be attached to any fixed object such as a shelf or mantelpiece, and the lamp thereby allowed to concentrate its light on any desired object. The lamp itself is fitted with two condensing lenses of suitable focal length, and the burner supplied with acetylene gas, which is generated from a tin cartridge contained in an appropriate receiver at the bottom of the lamp. A small water reservoir, when the tap is turned, allows drops of water to trickle slowly on to the carbide contained in the cartridge. Each cartridge will supply gas for about twenty minutes, and afford illumination sufficient for ordinary clinical examination. The maximum efficiency of the lamp is said to be about 25 candles. In our experimental trial of the lamp we hardly think we obtained this degree of luminosity, but we could obtain a good view of the larynx by its means. This little lamp should be very convenient for travelling, since it is compact and packs up into a very small space; and it is perfectly safe and free from danger if the directions are intelligently carried out.

The toilette requisites—namely, the Birch Tar and Sulphur Soap, as well as the Emollient Bath Powder—are excellent examples of the specialities for which this firm have obtained a well-deserved reputation. Each of them is a preparation of careful manufacture and fine finish.

